# deltaNp63 Has a Role in Maintaining Epithelial Integrity in Airway Epithelium

**DOI:** 10.1371/journal.pone.0088683

**Published:** 2014-02-12

**Authors:** Ari Jon Arason, Hulda R. Jonsdottir, Skarphedinn Halldorsson, Berglind Eva Benediktsdottir, Jon Thor Bergthorsson, Saevar Ingthorsson, Olafur Baldursson, Satrajit Sinha, Thorarinn Gudjonsson, Magnus K. Magnusson

**Affiliations:** 1 Stem Cell Research Unit, Biomedical Center, Faculty of Medicine, University of Iceland, Reykjavik, Iceland; 2 Faculty of Pharmaceutical Sciences, University of Iceland, Reykjavik, Iceland; 3 Department of Pharmacology & Toxicology, Faculty of Medicine, University of Iceland, Reykjavik, Iceland; 4 Center for Systems Biology, University of Iceland, Reykjavik, Iceland; 5 Department of Laboratory Hematology, Landspitali University Hospital, Reykjavik, Iceland; 6 Department of Pulmonary Medicine, Landspitali University Hospital, Reykjavik, Iceland; 7 Department of Biochemistry, Center for Excellence in Bioinformatics and Life Sciences, State University of New York at Buffalo, United States of America; 8 Institute of Immunobiology, Kantonal Hospital, St. Gallen, Switzerland; Cincinnati Children’s Hospital Medical Center, United States of America

## Abstract

The upper airways are lined with a pseudostratified bronchial epithelium that forms a barrier against unwanted substances in breathing air. The transcription factor p63, which is important for stratification of skin epithelium, has been shown to be expressed in basal cells of the lungs and its ΔN isoform is recognized as a key player in squamous cell lung cancer. However, the role of p63 in formation and maintenance of bronchial epithelia is largely unknown. The objective of the current study was to determine the expression pattern of the ΔN and TA isoforms of p63 and the role of p63 in the development and maintenance of pseudostratified lung epithelium *in situ* and in culture. We used a human bronchial epithelial cell line with basal cell characteristics (VA10) to model bronchial epithelium in an air-liquid interface culture (ALI) and performed a lentiviral-based silencing of p63 to characterize the functional and phenotypic consequences of p63 loss. We demonstrate that ΔNp63 is the major isoform in the human lung and its expression was exclusively found in the basal cells lining the basement membrane of the bronchial epithelium. Knockdown of p63 affected proliferation and migration of VA10 cells and facilitated cellular senescence. Expression of p63 is critical for epithelial repair as demonstrated by wound healing assays. Importantly, generation of pseudostratified VA10 epithelium in the ALI setup depended on p63 expression and goblet cell differentiation, which can be induced by IL-13 stimulation, was abolished by the p63 knockdown. After knockdown of p63 in primary bronchial epithelial cells they did not proliferate and showed marked senescence. We conclude that these results strongly implicate p63 in the formation and maintenance of differentiated pseudostratified bronchial epithelium.

## Introduction

Stratified epithelial tissues depend on somatic progenitor cells to maintain their integrity and homeostasis. It has been demonstrated that p63-positive basal epithelial cells serve as a source of other differentiated cells in stratified epithelial tissues including skin (reviewed in [1]). The epithelial layer of the upper airways is lined with a pseudostratified columnar epithelium. A fundamental difference between stratified and pseudostratified epithelia is that all cells of the pseudostratified epithelium are connected to the basement membrane i.e. differentiation does not confer loss of basement membrane adhesion.

Deregulation of repair mechanisms and cell differentiation in the bronchial epithelium are important factors in the pathogenesis of several lung diseases such as asthma, chronic obstructive pulmonary disease (COPD), pulmonary fibrosis and carcinoma [2], [3]. Understanding the molecular events regulating hierarchical differentiation and repair mechanisms in airway epithelia is therefore of major interest. p63 expressing basal cells of the mouse trachea and human bronchial tree have been identified as potential progenitor cells during lung development and epithelial repair. This has been shown by different experimental approaches including lineage tracing, injury infliction on the mouse lung and human *in vitro* culture models [4], [5]. Furthermore, p63 has been shown to regulate the expression of cytokeratin (CK) 14 which under normal circumstances is expressed in isolated clusters of basal cells in the bronchial epithelium but is upregulated during airway epithelial repair following injury [5]–[9]. It has been hypothesized that in the mouse lung, p63 positive basal cells give rise to early progenitor luminal cells positive for CK8 through a notch-dependent mechanism. Later, these cells differentiate terminally to ciliated, clara or goblet cells based on secondary notch signaling processes [10]. Other factors such as the cytokine interleukin 13 (IL-13) have been shown to affect goblet cell differentiation in the airways, with goblet cell hyperplasia reported to be a major contributing factor in diseases like asthma [11], [12].

The p63 protein belongs to the p53 family of transcription factors that also includes p53 and p73. It has been shown to regulate the epithelial differentiation program, especially in stratified squamous epithelia such as skin. Unlike the uniform expression of its relative p53, p63 is mostly restricted to basal cells of diverse stratified epithelia including skin, prostate and breast. Functional characterization of the p63 gene is complicated by alternative promoter usage and/or splicing events leading to the generation of at least ten different isoforms. These isoforms can be divided into two major groups, i.e. TA-p63 and ΔN-p63. Their transcription is driven from separate promoters (P1 and P2) generating the TA-p63 isoform containing a transactivating (TA) domain at the N-terminus (P1) or the ΔN-isoform (P2) which does not have a functional transactivating domain. Both types of isoforms can then further be alternatively spliced at the carboxy terminus in five different ways (α, β, γ δ and ε) generating a total of ten different isoforms [13]–[16] (reviewed in [1]).

Mice lacking p63 die postnatally due to multiple developmental defects, including abnormalities in stratified tissues such as the skin, breast and prostate [17]–[19].

Initial reports on the p63-knockout phenotype raised fundamental questions regarding the role of p63 in regulating the epithelial differentiation program. Although two independent studies initially generating p63-knockout mice described similar phenotypic observations such as epithelial disruption, abnormal limb and craniofacial development, their conclusions on the role of p63 differed [17], [18]. Mills et al. argued that p63 was essential for the commitment of simple ectoderm to epidermal lineages [18], whereas Yang et al. suggested a role in maintaining the proliferative potential of epithelial stem cells [15], [17].

In most basal epithelial cells the predominant isoforms are of the ΔNp63 type and in adult epithelium these are believed to be master regulators of epithelial differentiation in stratified epithelium [20]. Recently, the ΔNp63 isoform was specifically silenced in adult epithelial tissues revealing a major role for ΔNp63 in epithelial stratification and skin stem cell renewal [21].

Although many studies have provided insight into the role of p63 in the regulation of stratification in the skin, much less is known about the role of p63 in the airway epithelium. p63−/− newborn mice lack basal cells in the tracheobronchial epithelium and the pseudostratified epithelium in the upper airways is replaced by a simple epithelial lining [22]. On the other hand, overexpression of ΔNp63 in the mouse lung may result in squamous metaplasia of the alveolar region [7]. Furthermore, ectopic expression of TAp63 can induce metaplastic lesions in simple lung epithelia [23]. The importance of ΔNp63 is highlighted in a recent publication of the Cancer Genome Atlas Research Network aimed at identifying critical gene alterations in squamous cell lung cancer. The study underlined the importance of genes involved in squamous cell differentiation (mutated in 44% of samples) including SOX2 and ΔNp63 [24]. Although there is still controversy regarding roles of specific isoforms of p63, it is clearly important for stratification and stem cell biology of the airway epithelium.

We have recently established a human bronchial epithelial cell line (VA10) with basal cell characteristics [25]–[28]. In conventional 2-D monolayer culture this cell line maintains a predominant basal cell phenotype, expressing p63 and other basal markers such as CK5 and −14. Interestingly, when VA10 is cultured under air-liquid interface (ALI) conditions on transwell filters, the cells undergo a differentiation process forming a functional bronchial layer, with mature tight junctions. Under these conditions, the VA10 epithelium contains both undifferentiated p63 expressing cells in a basal position, and differentiated p63-negative cells towards the luminal/air side mimicking the normal tracheobronchial epithelium [25]. In this study we used this basal cell model to study the role of p63 in generating and maintaining pseudostratified bronchial epithelium *in vitro* and to test if p63 is necessary for terminal differentiation of cells in the bronchial epithelium.

## Materials and Methods

### Ethics Statement

Lung tissue samples were provided by written informed consent from patients undergoing lung surgical procedures. This procedure has been approved by the Landspitali Hospital Ethics Committee. Reference number 88374–96345.

### Cell Culture

The bronchial epithelial cell line VA10 was previously established at the laboratory [25]. The VA10 cell line is available upon request for all academic investigators for non-commercial purposes. Cells were cultured in LHC9 medium (Invitrogen, NY, USA) supplemented with 50 IU/ml penicillin and 50 µg/ml streptomycin (Invitrogen). For air-liquid interface cultures, cells were seeded on the upper layer of Transwell cell culture filters (Corning®Costar®) pore size 0.4 µm, 12 mm diameter, polyester membrane) (Sigma-Aldrich, St. Louis, USA) at density of 2×10^5^ cells per well. The cultures were maintained on LHC9 for 5 days, 0,5 ml in the upper chamber and 1.5 ml in the lower chamber. After 5 days medium was changed to DMEM/F-12 (Invitrogen), supplemented with 2% Ultroser G (Cergy-Saint-Christophe, France) for another 5 days. For air-liquid interface culture, the medium was aspired from the apical side and rinsed with PBS. Primary cells were isolated from patient material with enzyme digestion and cultured in chemically defined bronchial cell growth medium as previously described [29] Isolated cells were maintained on collagen coated flasks (VitroCol, Advanced BioMatrix) and cultured in chemically defined bronchial epithelial cell medium (BEGM, Life Technologies/Sigma) as previously described [30].

### Production of Lentivirus and Cell Transduction

For production of lentivirus containing scrambled hairpin (pLKO.1 shSCR) (Addgene plasmid 17920) [31] or shRNA against p63 (shp63alpha pLKO.1 puro) (Addgene plasmid 19120) [32], general online guidlines for pLKO.1 vectors from addgene.com were followed with modifications. Arrest-in (Open Biosystems) was used according to the manufacturer’s specifications, instead of the recommended FuGENE. 70% confluent HEK-293T cells were cultured for 24 h w/o antibiotics and transfected with lentiviral transfection constructs and packaging plasmids (psPAX2 and pMD2.G) (Addgene plasmids 12260 and 12259, respectively). Culture medium containing the virus was harvested 24 and 48 hours post transfection and centrifuged at 1250 rpm at 4°C for 5 minutes and filtered through 0,45 µm filter.

Transduction of cells was done as indicated in guidlines for pLKO.1 vectors from addgene.com. Lentiviral particle solution was added to culture medium (containing 8 mg/ml polybrene) of 70% confluent cells at low MOI volume (70 µl per 6 cm target plate) and incubated for 20 hours. Cells were then cultured for 24 hours on fresh culture media. Infected cells were then selected with puromycin at a final concentration of 0,7 µg/ml, which according to titration assays was determined sufficient to eliminate uninfected cells in 48 hours.

### Immunochemistry

Paraffin embedded tissue samples of normal lung biopsies were obtained from the Department of Pathology, Landspitali University Hospital. The samples were deparaffinized, refixed in methanol/acetone (1/1) for 5 minutes at −20°C and stained with EnVision®+ System-HRP kit (Dako). Primary antibodies (ncl-p63 (clone 7JUL) from *Leica Microsystems* and ΔNp63, provided by Dr. Sat Sinha, (University of Buffalo, NY) were incubated overnight at 4°C. Cell cultures were fixed in 3,7% formaldehyde. For nuclear protein staining, cells were also fixed in methanol/acetone (1/1) for 5 minutes at −20°C. They were then washed two times for 10 minutes at room temperature with PBS, and blocked with 10% goat serum in IF-buffer (0.2% Triton X-100; 0.1% BSA and 0.05% Tween-20 in PBS). Primary antibodies (ncl-p63 (clone 7JUL)) and ΔNp63, (RR-14, [7]), were incubated overnight at 4°C, followed by three 10 minute washes in PBS. Isotype specific secondary antibody conjugates Alexa Fluor® (Invitrogen) were incubated for 2 hours at RT. After PBS rinsing, nuclear staining was performed with TO-PRO-3® (Invitrogen) for 30 minutes followed by 3×10 minute washes. The samples were then embedded in Fluormount-G (Southern Biotech, Birmingham, AL) for microscopic analysis.

### Microscopy

Immunofluorescence was visualized and captured using laser scanning Zeiss LSM 5 Pascal Confocal Microscope (Carl Zeiss AG, Munich, Germany). Bright-field and phase-contrast images of Matrigel cultures were captured using a Leica DFC320 digital camera attached to a Leitz Fluovert microscope (Wetzlar, Germany).

### Assessment of Cellular Senescence

A commercially available kit for detection of senescense associated β-galactosidase (Cell Signaling, #9860) was used according to the manufacturer specifications. After fixation, cells were incubated with staining solution overnight at 37°C. Ten representative images were taken of each flask. CellProfiler (cellprofiler.org) was used to measure area occupied by blue stained cells. A pipeline was made to correct for illumination difference within each image, after which the image was thresholded to give a binary image where blue stained cells appeared white on a black background. White areas were then measured as a ratio of total area. Data is represented as a ratio of β-galactosidase levels compared with control cells (VA10^Scr^).

### Transwell Migration

50.000 starved cells were seeded in DMEM/F12 basic medium on Falcon® (BD) cell culture inserts with 8 µm pore size in triplicates. LHC9 medium (Invitrogen) was used as a chemoattractant in the lower chamber. After 12 h incubation, cells in upper chamber were removed with a cotton swab and migrated cells in lower chamber fixed with 3,7% formaldehyde, stained with 0,1% crystal violet, rinsed with PBS and counted.

### Proliferation Assay

20.000 cells per well were seeded onto 12 well culture plates (Falcon®, BD). Each day, three wells from each culture were fixed with 3,7% formaldehyde, rinsed and stained with 0,1% crystal violet for 15 min. After staining, the cells were rinsed again with PBS. After the culture period, the crystal violet was released using acetic acid. Absorbance was measured at 590 nm.

### Transepithelial Electrical Resistance (TEER) Measurements

TEER of ALI cultured VA10 cell layers was measured with Millicell-ERS volthometer (Millipore, MA, USA). Prewarmed PBS was added to the apical side of ALI cultured cells to be able to measure the TEER. The corrected TEER value was obtained after subtraction of the background from the cell-free culture insert.

### Permeation Studies

ALI cultured VA10 cell layers were used in the sodium fluorescein (Flu-Na) (Sigma-Aldrich) permeation studies to determine the permeability of the paracellular epithelial barrier. Prior to the permeation studies, the cell layers were washed twice with prewarmed Hanks Balanced Salt Solution (HBSS) and allowed to equilibrate in the incubator for 30 min in HBSS (0.5 mL apical and 1.5 mL basolateral). Flu-Na was dissolved in HBSS buffer to a final concentration of 50 µM. Before the permeation studies were started, the HBSS buffer was aspirated from the apical surface and replaced by 0.52 mL of prewarmed HBSS test solution, with 1.50 mL of HBSS in the basolateral chamber. Immediately, a sample (20 µL) of the test solution was removed to determine the initial concentration (C_0_). The cells were incubated at 37°C and agitated on an orbital shaker at 80 rpm. Sampling from the basolateral compartment (100 µL) was done after 20, 40, 60, 80 and 120 min and replaced with equal volume of fresh HBSS buffer.

Samples from Flu-Na studies were directly added to black 96 well plates and 10 mM NaOH aqueous solution (100 µL) was added to each sample. Fluorescence was measured with excitation wavelength of 485 nm and emission of 535 nm using a fluorospectrometry (Tecan GENios microplate reader). Apparent permeability (P_app_) coefficients were calculated from the Flu-Na permeation experiment according to equation 1, where dQ/dt is the flux, A is the surface of the cell filter (1.12 cm^2^) and C_o_ is the initial concentration in donor chamber. 
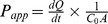
.

### q-PCR

RNA was isolated using Tri Reagent® solution (Ambion) and cDNA preparation was carried out using RevertAid™ First strand cDNA Synthesis Kit (Fermentas) according to the manufacturer’s instructions. Real-time PCR using Power SYBR Green PCR Master mix (Applied Biosystems) was used to detect the relative quantity of Muc5ac. The following primers were used for Muc5ac; fwd: 5′-ggcgatgatgaagaaggttga-3′, rev: 5′-gacactgagcctggatgg-3′ (Integrated DNA Technologies, IDT). Tubulin was used as the endogenous reference gene and amplified using the following primers; fwd: 5′-cctccttccgtaccacatc-3′, rev: 5′-gccagatctttagaccagacaa-3′ (IDT). For quantification of specific isoforms of p63 we used a labeled probe real-time PCR assay based on the following primers; TAp63 fwd: 5′-gtgcgacaaacaagattgagattag-3′, Tap63 rev: 5′-tgttcaggagccccaggtt-3′. ΔNp63 fwd: 5′-aaaggacagcagcattgatcaa-3′, ΔNp63 rev: 5′- tgttcaggagccccaggtt-3′ (TAG Copenhagen). The fluorochrome labeled probes were as follows; TAp63; 5′-Fam-acctgagtgaccccatg-BHQ-1-3′, ΔNp63; 5′-Hex-cttacagctaacatgttgtacc-BHQ-1-3′ (TAG). GAPDH was used as the endogenous reference gene and amplified using commercially available primers from Applied Biosystems (part number 4326317E).

For evaluation of p63 knockdown in primary bronchial cells; real-time PCR using Light Cycler fast start DNA master plus SybrGreen I (Roche) was used to detect the relative quantity of p63. Cycle profile, 10 min at 95°C; 45 cycles of 10 s at 95°C, 20 s at 50°C, and 20 s at 72°C; followed by a melting curve step to confirm product specificity. Relative gene expression was calculated using the 2^−ΔΔ*Ct*^ method. Endogenous control was 18S rRNA based on the following primers fwd: 5′-gctggaattacccgcggct-3′, rev: 5′-cggctaccacatccaaggaa-3′ (Microsynth, Switzerland).

### Wound Healing Assay

VA10^Scr^ and VA10^p63kd^ were cultured to confluence in 2-well chamber slides (BD). Confluent monolayers were scratched with a P200 pipette tip to inflict wounds. Live cell imaging was performed on a Leica DMI6000B inverted microscope, fitted with an incubation chamber and local CO_2_ control. Automated imaging of 4 locations for each cell line was performed at 2 minute intervals. Wound healing was estimated at 2,4,6,8 and 10 hours post scraping using CellProfiler image analysis software [33].

### IL-13 Induction

Cells were cultured for 5 days on LHC-9 and then for 5 days on DMEM/UG in a submerged culture. After 5 days of ALI culture, IL-13 (Peprotech, London, UK) was added to basal side to a final concentration of 25 ng/ml and cultured for 14 days.

### Apoptosis Assay

Cells were harvested and washed twice with cold PBS and resuspended in 1x binding buffer at concentration 1×10^6^ cells/ml. Then 100 µl of the solution (1×10^5^ cells) were transferred to a 15 ml centrifuge tube. 5 µl of FITC Annexin V (BD Pharmingen) were added to the cells and incubated at RT in the dark for 30 minutes. 400 µl of 1x binding buffer were added to each tube and 5 µl of propidium iodide (PI) (BD Pharmingen) 10 minutes before at least 10.000 cells from each sample were analyzed in MACSQuant (Miltenyi).

### Statistical Analysis

Data are presented as means with standard deviations of measurements unless stated otherwise. Statistital differences between samples were assessed with Student two –tailed T-test. P-values below 0.05 were considered significant (***p≤0.001, **p≤0.01, *p≤.0.5).

## Results

### ΔNp63 is Expressed in Bronchial Basal Epithelial Cells in situ and in ALI Culture

**Figure 1:**
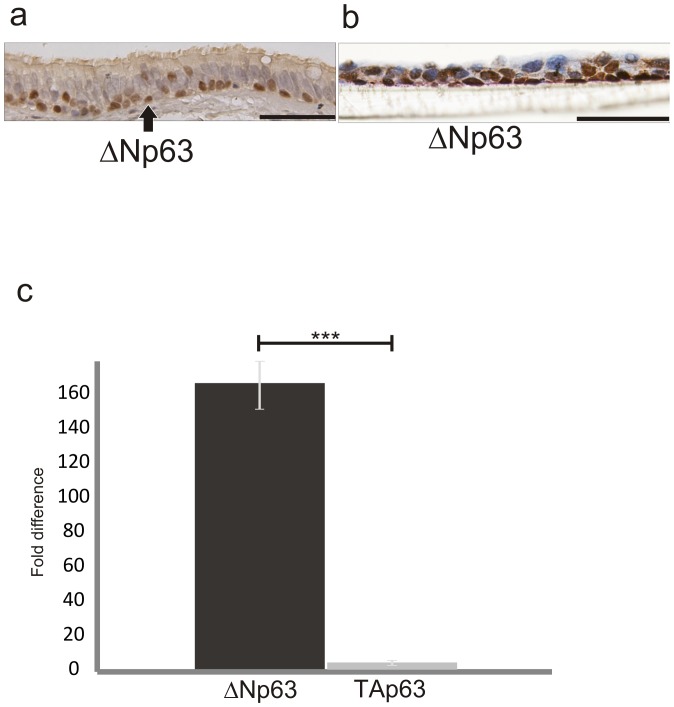
ΔNp63 is expressed in bronchial basal cells *in situ* and is a major p63 isoform in basal cells *in* *vitro*. ΔNp63 is expressed in basal cells in normal human bronchi (a). VA10 cells cultured in ALI express ΔNp63 at the basolateral side and not on the apical side (b). ΔNp63 shows 167,5 fold expression compared to TAp63 in bronchial basal cell line VA10, as calculated by ΔΔct obtained by qRT-PCR. GAPDH was used as endogenous control. (c). Scale bars 50 µm. ***p≤0.001.

ΔNp63 isoforms are proposed to be the most abundant isoforms of p63 in epithelial tissues [34]. These prominent isoforms have been shown to be expressed in basal epithelial cells in diverse epithelial organs where they are believed to be regulators of differentiation and stratification [20]. VA10 has been shown to generate bronchial epithelium in ALI culture [25]. To examine the expression pattern of p63 in the human lung we used a pan-p63 (non-isoform specific p63) and a ΔNp63 isoform specific antibody to stain a normal lung that was compared to the ALI cultured VA10 *in* *vitro* model. The ΔNp63 isoform specific antibody indicates expression in the basal layer of the human bronchial epithelium, in close proximity to the basement membrane (Fig. 1a). This pattern of expression is identical to staining with a non-isoform specific p63 antibody on duplicate samples, where only basal cells stain positive (Fig. S1a). In VA10-ALI culture, the staining closely mimics this expression pattern observed in the normal lung, with ΔNp63 isoform expression in nuclei located basally in the culture while nuclei lining the apical side were negative (Fig. 1b, Video S1). This pattern of expression is identical to staining with a non-isoform specific p63 antibody in VA10 ALI cultures (Fig. S1b). A quantitative real-time PCR (q-PCR) comparing the expression of the ΔNp63 isoform to the TA-p63 isoform in the ALI model further indicated that the ΔNp63 isoform is the major p63 isoform in VA10 epithelium (Fig. 1c).

### Knockdown of p63 Affects Viability and Phenotype of Bronchial Basal Cells

**Figure 2:**
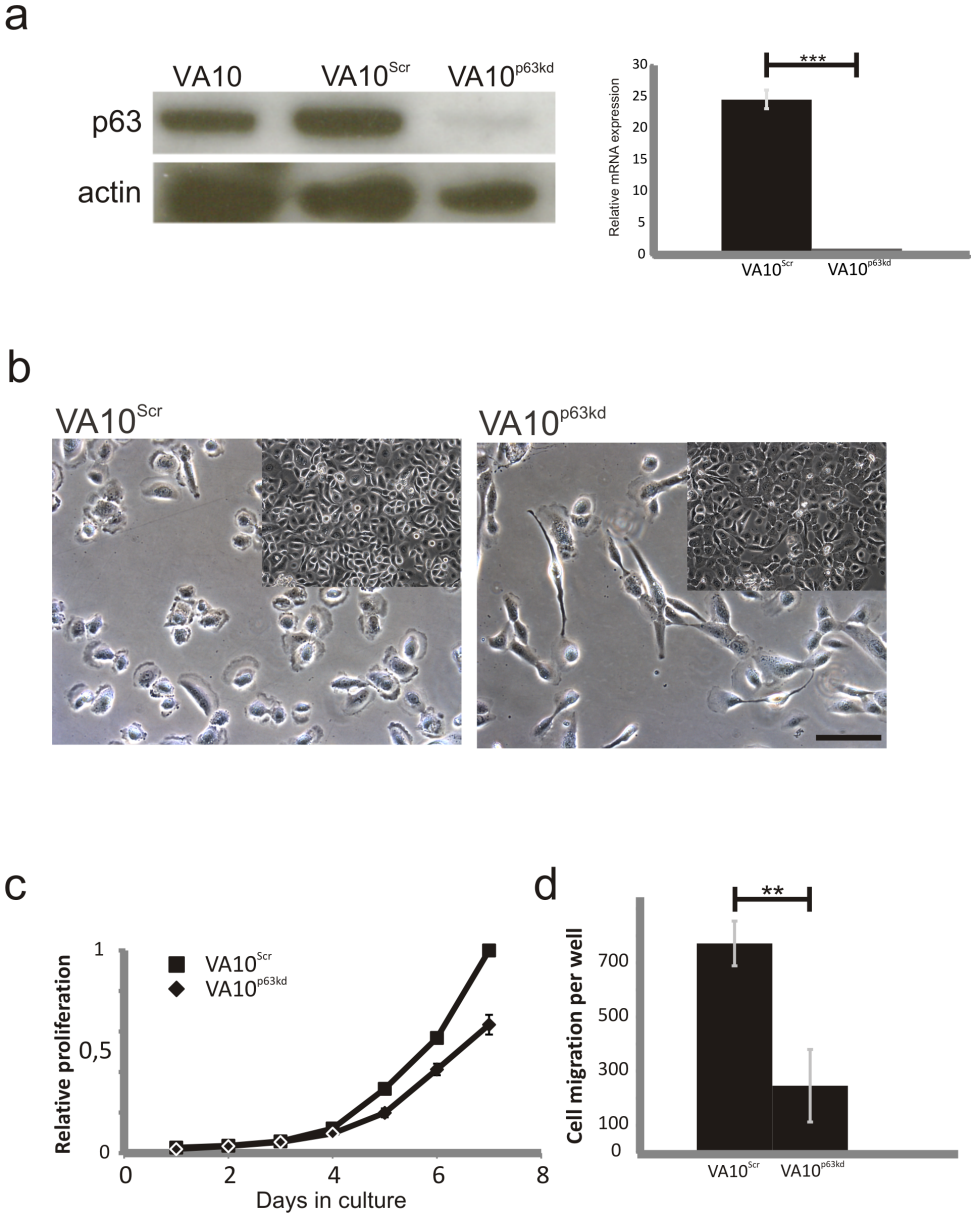
Knockdown of p63 affects cellular morphology, proliferation and migration. Western blot showing lentiviral siRNA knockdown of p63 in VA10 (a). A subset of VA10^p63kd^ (KD) cells obtains an elongated morphology in monolayer culture (b). A proliferation assay reveals 40% decrease in proliferation of KD cells compared to scrambled. Error bars represent 95% confidence intervals (c). A transwell migration assay shows reduced ability of KD cells to migrate through the filter (d). ***p≤0.001, **p≤0.01.

To test the functional significance of p63 in bronchial epithelial cells, we performed a lentiviral based knock-down of p63 in primary bronchial epithelial cells and the VA10 cell line (VA10^p63kd^). Using this approach, we saw a 71,5% knockdown of p63 in primary bronchial epithelial cells (Fig. S2a). However, p63-knockdown cells were unable to proliferate further and stained strongly with senescence-associated β-galactosidase (Fig. S2b and S2c, respectively). In VA10 knockdown cells, we found a significant downregulation of p63 protein levels by western blotting (Fig. 2a left) and a corresponding 25-fold reduction in p63 mRNA expression by q-PCR (Fig. 2a right). The knockdown of p63 in VA10 cells led to phenotypic changes in a subset of cells that became more elongated (Fig. 2b). This elongated phenotype disappeared when cells became confluent (Fig. 2b, insert). The VA10^p63kd^ cells showed decreased expression of the bronchial basal cell marker CK5/6 but not the basal cell marker CK17. Expression of CK14, a marker of reactive bronchial epithelium was diminished, as was the expression of the type III intermediate filament vimentin. Expression of the pan-epithelial differentiation antigen EpCAM was increased in VA10^p63kd^ (Table 1 and Fig. S3). To assess if p63 plays a role in proliferation of bronchial basal cells, 20,000 cells were seeded on each plate and their proliferation was monitored over a period of 7 days. By the 5^th^ day of cell culture, VA10^p63kd^ proliferated significantly less compared to scrambled control (VA10^Scr^) and this difference increased on the following days. On day seven, the crystal violet absorbance from VA10^p63kd^ cells was 60% compared to VA10^Scr^ (fig. 2c).

**Table 1:** Expression of selected epithelial markers in VA10^Scr^ and VA10^p63kd^.

Marker	VA10^Scr^	VA10^p63kd^
CK5/6	+*	+/−
CK14	+	−/+
CK17	+	+/−
EpCam	−/+	+/−
Vimentin	+	+/−

−, negative; −/+ predominantly negative; (+) weakly positive; +/−, predominantly positive; +, positive. *Few negative cells.

To test the effects of the p63 knockdown on cellular migration 50,000 VA10^Scr^ and VA10^p63kd^ cells were seeded, respectively, on a filter with 8,0 µm pore size with a nutrient-rich medium placed in the lower chamber. VA10^p63kd^ showed markedly reduced migratory properties in this assay compared to scrambled control, with 226(+/−130 SEM) cells/well migrating through the filter after 24 h, compared to 772(+/−83) VA10^Scr^/well (fig. 2d).

**Figure 3:**
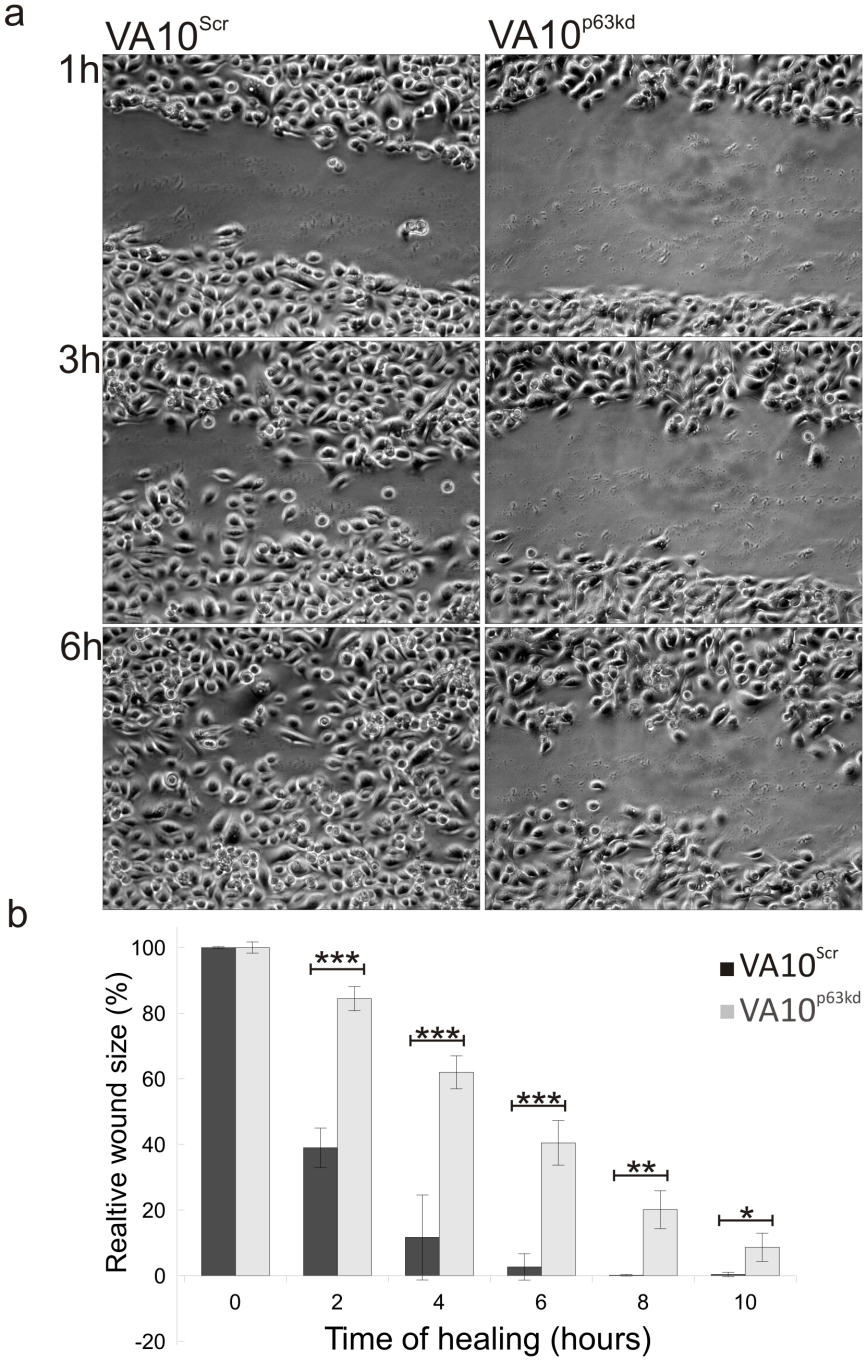
p63 is necessary for a quick wound healing response. Wound healing assay shows decreased healing capacity of VA10^p63kd^ cells (right) compared to VA10^Scr^ cells (left) after 1,3 and 6 hours (a). Images represent results obtained from 4 independent experiments. Graph showing relative wound healing speed between VA10^p63kd^ cells and VA10^Scr^ cells (b). ***p≤0.001, **p≤0.01, *p≤.0.5.

These observations led us to hypothesize that VA10^p63kd^ cells had reduced wound healing properties. To test this, we performed wound healing assays where confluent monolayers of VA10^p63kd^ cells and VA10^Scr^ cells were scratched with a pipette tip and wound healing measured (Fig. 3a). The VA10^p63kd^ cells had a markedly slower repair response compared to the control VA10^Scr^ cells. VA10^Scr^ fully repaired the wound in approx. 6 h while the VA10^p63kd^ cells repaired the wound in approx. 10 h (Fig. 3b, Videos S2 and S3).

**Figure 4:**
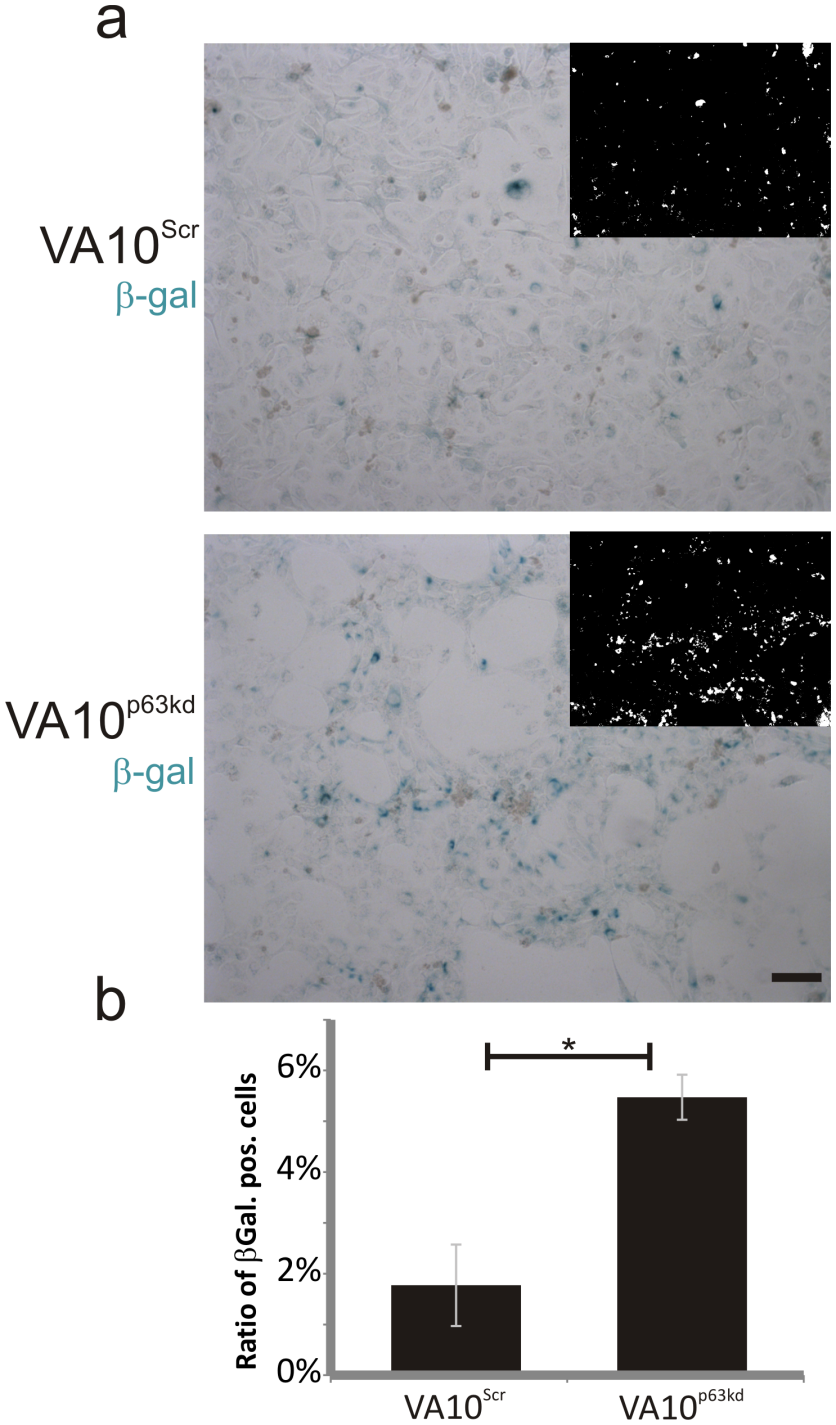
VA10^p63kd^ cells lose survival ability. VA10^p63kd^ (KD) cells enter senescence when cultured for prolonged period in monolayer, as shown with β-galactosidase staining (a). To quantify the senescence the pixel intensity is represented in bars for VA10^Scr^ compared to VA10^p63kd^. Scale bars 50 µm. *p≤.0.5.

p63 has been linked to cell survival and been reported to help cells bypass cellular senescence [35]. To test this in lung basal cells, we cultured VA10^scr^ and VA10^p63kd^ over a prolonged period. After reaching confluence, the cells were further cultured for 14 days. By that stage, a substantial number of VA10^p63kd^ had detached from the monolayer. To determine whether the VA10^p63kd^ cells were undergoing senescence, the cultures were stained with β-galactosidase (Fig. 4a). A three-fold increase in β-galactosidase activity, as measured by pixel intensity, was observed (Fig. 4b) suggesting that VA10^p63kd^ are predisposed to senescence. To test if cells were senescent earlier in culture, we stained early confluent cell cultures. A minor population of cells stained positive. Compared, VA10^p63kd^ cells showed 35% increase in positive cells to VA10^Scr^ (Fig. S4). We then set out to address if the loss of cells was due to apoptosis. Confluent cultures of VA10^Scr^ and VA10^p63kd^ cells were analyzed with flow cytometry using Annexin V and PI stainings, but increase in apoptosis of VA10^p63kd^ cells was not observed (Fig. S5).

### Loss of p63 Affects Epithelial Integrity and Impairs Stratification

**Figure 5:**
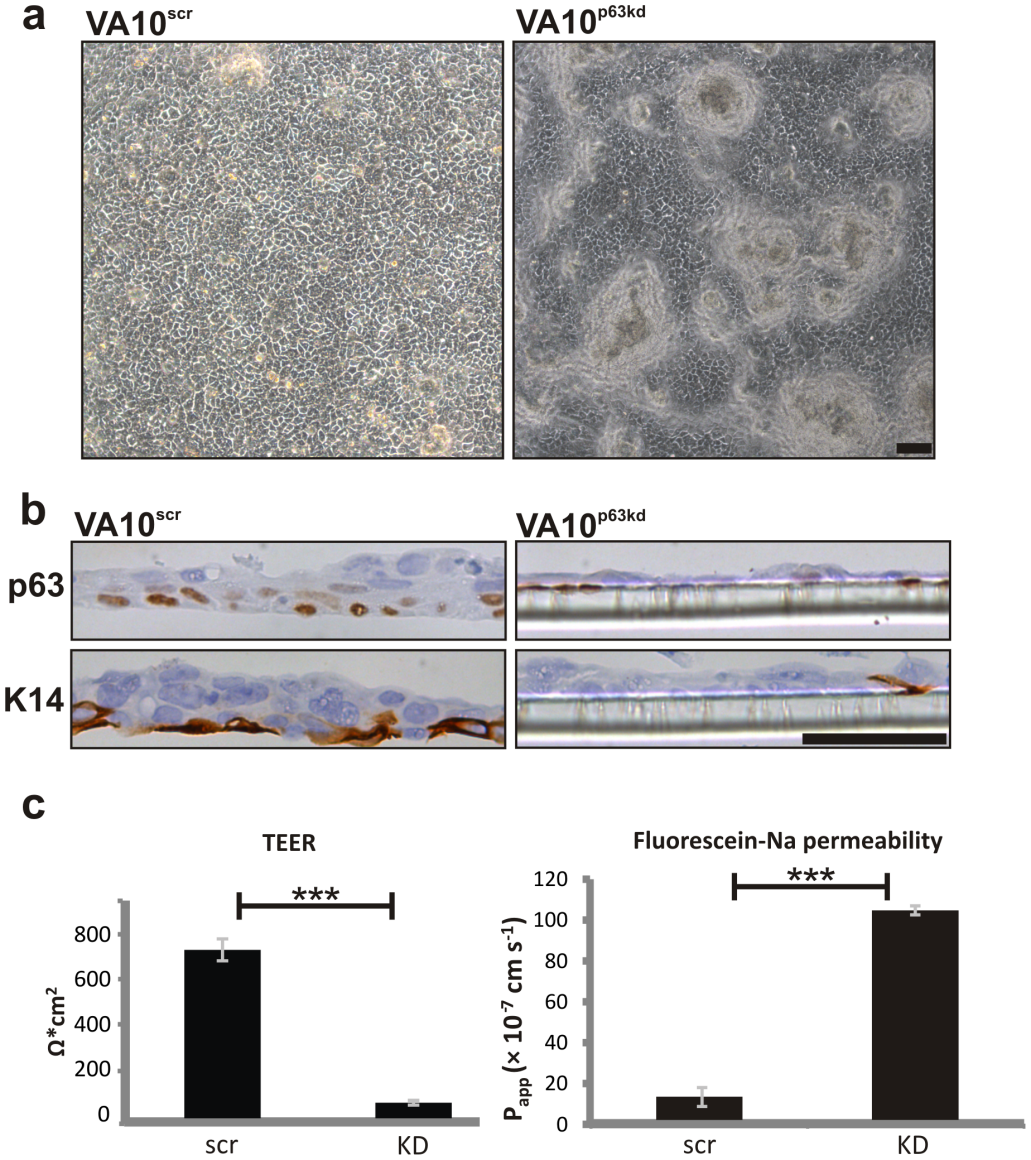
VA10^p63kd^ form impaired lung epithelium in ALI culture. VA10^p63kd^ cells form simple epithelium with random epithelial budding (7 days after initiation of ALI) (a) and significant downregulation of CK14 (14 days after initiation of ALI) (b). The VA10^p63kd^ epithelium does not form transepithelial electrical resistance (c) and has high permeability of Flu-Na (d) compared to scrambled control (14 days after initiation of ALI). Scale bars 50 µm. ***p≤0.001.

VA10 cells were cultured under ALI to study the involvement of p63 in bronchial epithelial integrity. VA10 cells in ALI culture generate a pseudostratified-like bronchial epithelium with high transepithelial electrical resistance (TEER) [25]. To test the functional role of p63 in generating intact pseudostratified-like epithelial lining, we compared the VA10^p63kd^ cells to their counterpart (scrambled siRNA) in ALI culture. After 2 weeks of ALI initiation, the epithelium in the VA10^p63kd^ cells was morphologically different with the lining predominantly forming a simple non-stratified epithelium (Fig. 5a and 5b) while clusters were seen as soon as one week of ALI, where cells formed irregular stratification. In these clusters, p63 expression was evident in the basal layer (Fig. S6). There was marked downregulation of CK14 in the VA10^p63kd^ cells (Fig. 5b). Furthermore the integrity of VA10^p63kd^ epithelia was disrupted as evidenced by lack of measurable TEER, compared to the control cells treated with scrambled siRNA that generated a resistance of 740 Ω*cm^2^ after 16 days in culture (Fig. 5c). In addition, the knockdown epithelium was highly permeable to the paracellular permeation marker Flu-Na compared to the scrambled control (Fig. 5c). Due to the marked effects of p63 knockdown on proliferation in primary bronchial epithelial cells we were not able to test them in the ALI culture model.

### VA10^p63kd^ Cells Fail to Show Goblet Cell Differentiation after IL-13 Stimulation

**Figure 6:**
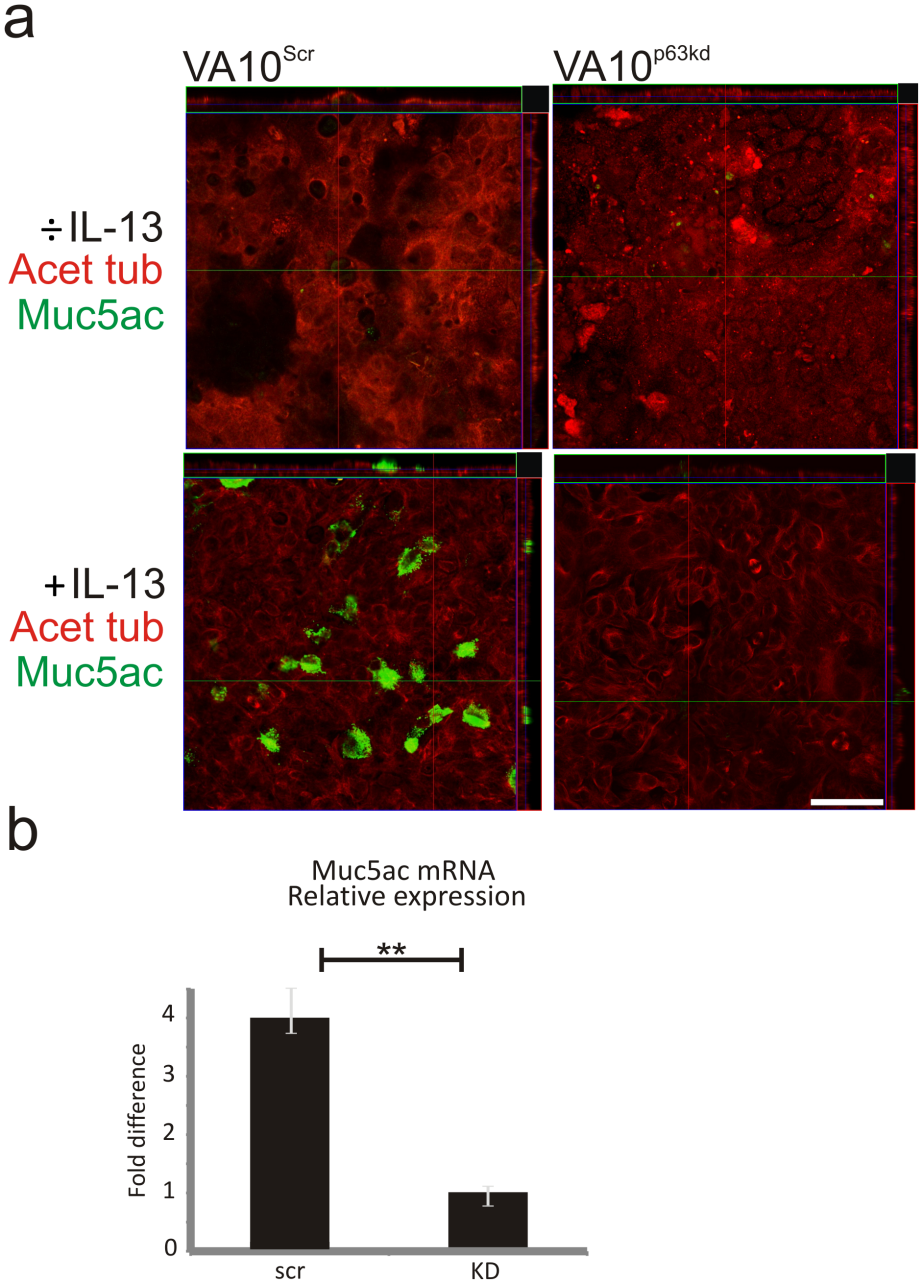
p63 is necessary for IL-13 induced goblet cell differentiation in ALI culture. A subset of VA10^Scr^ differentiates to goblet cells when stimulated with IL-13 (left panel). VA10^p63kd^ cells are unable to form goblet cells when stimulated with IL-13 (right panel). Scale bars 50 µm. **p≤0.01.

It is well documented that interleukin-13 (IL-13) is an inducer of goblet cell differentiation in both human and murine airways and this IL-13 induced differentiation has been suggested to explain the goblet cell hyperplasia in asthma [11], [12]. In the conventional ALI culture model, the VA10^Scr^ cells form ciliated cells as demonstrated by acetylated tubulin staining (Fig. 6). Addition of IL-13 to these culture conditions stimulates goblet cell differentiation in the control VA10^Scr^ cells resulting in a mixed population of ciliated (acetylated tubulin positive) and goblet cells (Muc5ac positive) (Fig. 6). When VA10^p63kd^ ALI epithelium was stimulated with IL-13, no goblet cell differentiation was observed. (Fig. 6).

## Discussion

In this study, we show that p63 is an important factor in the regulation of human bronchial epithelial integrity. We demonstrate that the ΔNp63 isoform is expressed in basal cells of the human bronchi, and is the major isoform expressed in an *in vitro* model of pseudostratification using VA10 basal cells in an ALI culture. Furthermore, knockdown of p63 in VA10 basal cells decreased cell proliferation, migration and wound healing properties. It also affected cell survival by facilitating cellular senescence in confluent culture. Furthermore, knockdown of p63 in VA10 cells led to disrupted epithelial integrity in ALI culture by inhibiting pseudostratification. This was accompanied with loss of TEER and increased paracellular permeability. We also observed suppressed IL-13 induced goblet cell differentiation in the p63 knockdown cells. Knockdown of p63 in primary bronchial epithelial cells resulted in growth arrest. Taken together, p63 appears to play an important role in forming and maintaining the viability and integrity of the human bronchial epithelium.

p63 is a critical transcription factor expressed exclusively in basal cells in many organs [19], [22], [36]. Initial studies on the effects of p63 in a mouse models described phenotypes with squamous epithelial disruption and abnormal limb and craniofacial development [17], [18]. Furthermore, it has been shown that ΔNp63 is necessary to maintain the clonogenic and proliferative potential of the basal progenitor cells in stratified epithelial cells *in vitro* [37], [38], and inactivation of p63 results in increased expression of ink4a and Arf tumor suppressors and [39], thus linking p63 to the control mechanism of cellular senescence and apoptosis. The finding of the chromatin remodeling protein Lsh as a direct target of ΔNp63αand an essential mediator of senescence bypassing, further supports these claims [35]. Vanbokhoven *et al*. suggested that p63 affected proliferative capacity of cells by either controlling proliferation directly or by preventing cell death [1]. DeCastro *et al.* reported that induction of miR203 in breast epithelial cell lines leads to downregulation of ΔNp63 and induces slower proliferation rate and even cell cycle arrest [40]. These effects could then be reverted with forced expression of ΔNp63. Our data indicate that similar mechanisms are probably also in play in human basal bronchial cells and this might explain the growth halt of primary cells after p63 knockdown.

The role of p63 in the human lung epithelium has not been well studied. We found that knockdown of p63 led to elongation of a subset of basal cells, consistent with studies from Barbieri *et al.* that showed squamous cell lines lacking ΔNp63 to possess more spindle shaped morphology than those expressing ΔNp63 [41]. p63 has been shown to affect proliferation of cells *in vitro* *[38]*, [*42*]. The relationship between p63 and cell motility seems to differ between organs and whether the cells are of native or carcinogenic origin. Studies on keratinocytes and head and neck squamous carcinoma show that loss of p63 leads to increased migration [41], [43]. In the repairing cornea, the β and γ isoforms of ΔNp63 have been shown to be upregulated in proliferating and migrating limbal and corneal cells [44]. In our model, loss of p63 appears to result in decreased migrational properties of bronchial basal cells.

To test the effects of p63 on cellular differentiation, we used IL-13, a common inducer of goblet cell differentiation. VA10^p63kd^ cells were unable to form goblet cells compared to the scrambled control. IL-13 induced goblet cell hyperplasia is one of the major hallmarks of the respiratory epithelium in asthma [11] and our finding, that p63 knockdown cells are unable to respond to IL-13, suggests a role for this transcription factor in upper airway basal cell differentiation. This could be due to less responsiveness to IL-13 or that the p63 plays a role in maintaining the basal cell in progenitor state, as has been suggested in skin basal cells [15], [17].

In addition to normal epithelial differentiation, p63 might also play a role in lung carcinogenesis, especially squamous cell carcinoma. Under chronic exposure to environmental pollutants (e.g. smoking) the mucociliated pseudostratified epithelium undergoes squamous metaplasia and cellular hyperplasia. This reduces mucociliary clearance, further aggravating the disease process. The resulting metaplasia is postulated to be a precursor lesion for infiltrating squamous cell carcinoma. The precise cellular mechanisms explaining squamous hyperplasia and metaplasia are not known. Interestingly, amplification of the p63 locus (on chromosome 3q27) frequently occurs in squamous cell carcinomas [45]. Furthermore, a strong association is seen between expression of p63 and squamous metaplasia and squamous cell carcinomas, suggesting a role for p63 in these pathological processes [45]–[48]. The recent demonstration that ΔNp63 is amplified and overexpressed in human squamous lung cancer and along with SOX2 function as oncogenes in a squamous differentiation program highlights the importance of p63 in lung cancer [24]. Defects in the control mechanisms regulating the expression of p63 may promote the undifferentiated phenotype, proliferation, and/or inhibition of apoptosis and therefore may play a role in tumorigenesis [46], [49], [50].

## Conclusions

In summary, our data suggest a critical role for the transcription factor ΔNp63 in maintaining the pseudostratified epithelial lining of the upper airways. Basal epithelial cells lacking p63 have diminished proliferative and migratory ability and increased sensitivity to cellular senescence. Furthermore, our data suggest that p63 might have a role in IL-13 induced goblet cell differentiation.

## Supporting Information

Figure S1 p63 is expressed in basal cells of the bronchial epithelium and in an *in vitro* model. p63 (brown) is expressed in basal cells lining the basement membrane of the bronchial epithelium (a). It is also expressed basally (green) and not apically in VA10 epithelium cultured in an *in vitro* air-liquid interface model (b).
(TIF)

Figure S2 Primary bronchial cells enter growth arrest following knockdown of p63. Quantitative real time PCR shows a 71,5% knockdown of p63 in bronchial basal cells compared to scrambled vector (a). The p63 knock down cells did not proliferate when re-seeded following puromycin selection but cells infected with scrambled vector did (b). The surviving p63 knockdown cells stained positive for β-galactoside at day p10 (c).
(TIF)

Figure S3 Expression of selected epithelial markers following knockdown of p63 in VA10 cells. DAB staining on VA10^Scr^ and VA10^p63kd^ cells shows downregulation of CK5/6, CK14 and Vimentin, upregulation of ESA and no difference in CK17 expression. The data shown represent results from two independent experiments that yielded similar results.
(TIF)

Figure S4 Low portion of VA10^Scr^ and VA10^p63kd^ cells are senescent at early confluency. β-galactosidase staining reveals low levels of senescent cells at confluency of VA10^Scr^ (upper) and VA10^p63kd^ (lower) cells (a). When compared, VA10^p63kd^ cells show a 35% increase in senescence compared to VA10^Scr^ (b).Error bars represent standard error of the means. Scale bars 50 µm. ***p≤0.001.
(TIF)

Figure S5 Knockdown of p63 does not affect apoptosis in monolayer VA10 cells. FACS analysis of Annexin V and PI stainings on confluent monolayer VA10^Scr^ and VA10^p63kd^ cells show similar portions of cells in early (Annexin high, PI low) and late (Annexin hign, PI high) apoptosis. Inset numbers represent percentage of each population in the quadrants. The data shown are represent results from two independent experiments that yielded similar results.
(TIF)

Figure S6 Rare patches of p63 positive cells are found in VA10^p63kd^ epithelium. When VA10^p63kd^ cells are cultured in ALI culture, rare patches of p63 positive cells can be found. These patches are able to form stratification and apical cells are p63-negative.
(TIF)

Video S1 z-stack of VA10^p63kd^ ALI culture stained with ΔNp63 antibody. Expression is limited to the basal layer and not to the apical side.
(MP4)

Video S2 A representative video of VA10^Scr^ cell wound healing process.
(MP4)

Video S3 A representative video of VA10^p63kd^ cell wound healing process.
(MP4)
