# Supplementary figures and images for: deltaNp63 Has a Role in Maintaining Epithelial Integrity in Airway Epithelium
Source: PLoS One. 2014 Feb 12;9(2):e88683. doi: 10.1371/journal.pone.0088683 (PMC3922990; doi:10.1371/journal.pone.0088683)

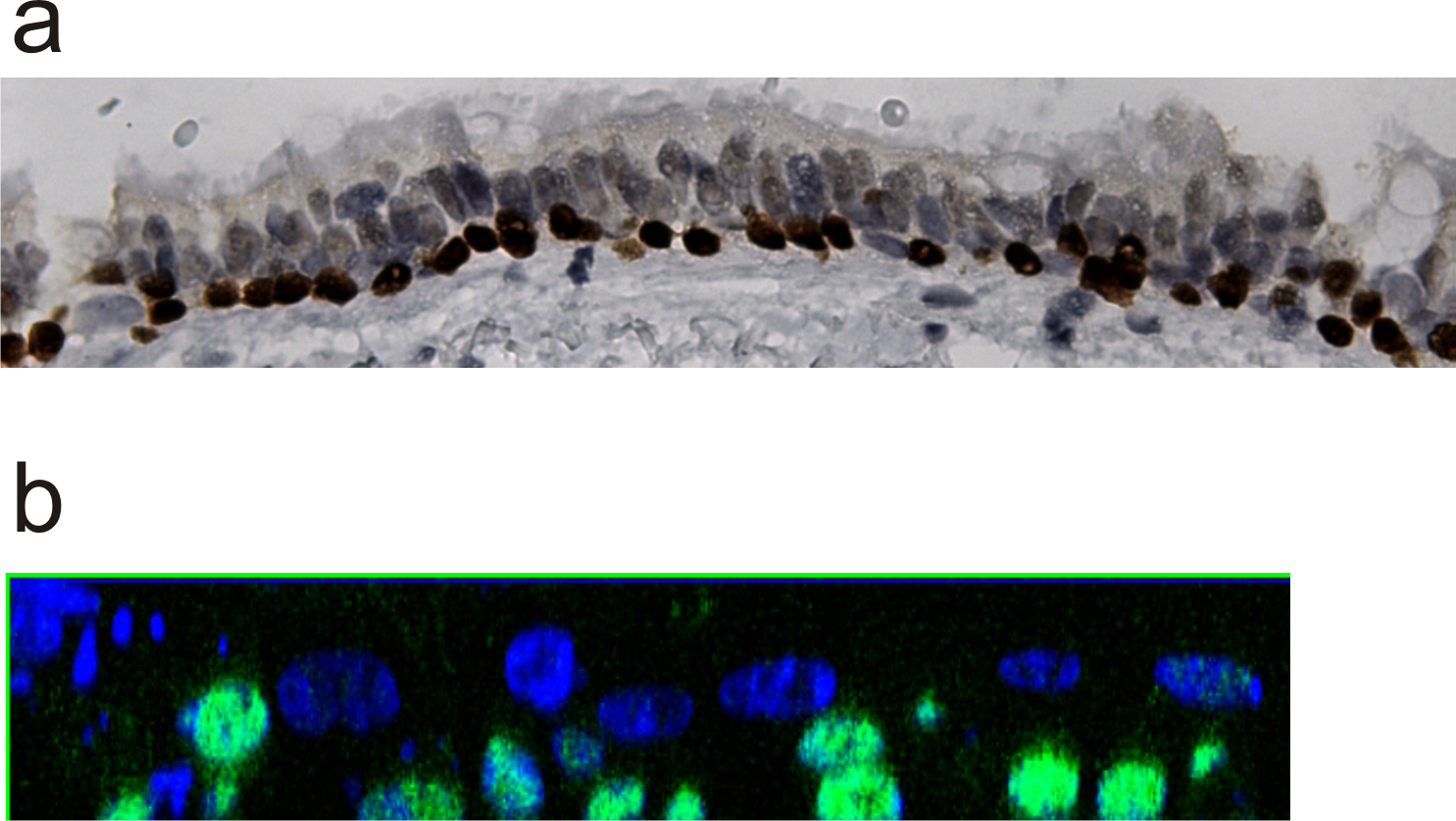

Supplement: Figure S1 — p63 is expressed in basal cells of the bronchial epithelium and in an in vitro model. p63 (brown) is expressed in basal cells lining the basement membrane of the bronchial epithelium (a). It is also expressed basally (green) and not apically in VA10 epithelium cultured in an in vitro air-liquid interface model (b). (TIF) [file pone.0088683.s001.tif]

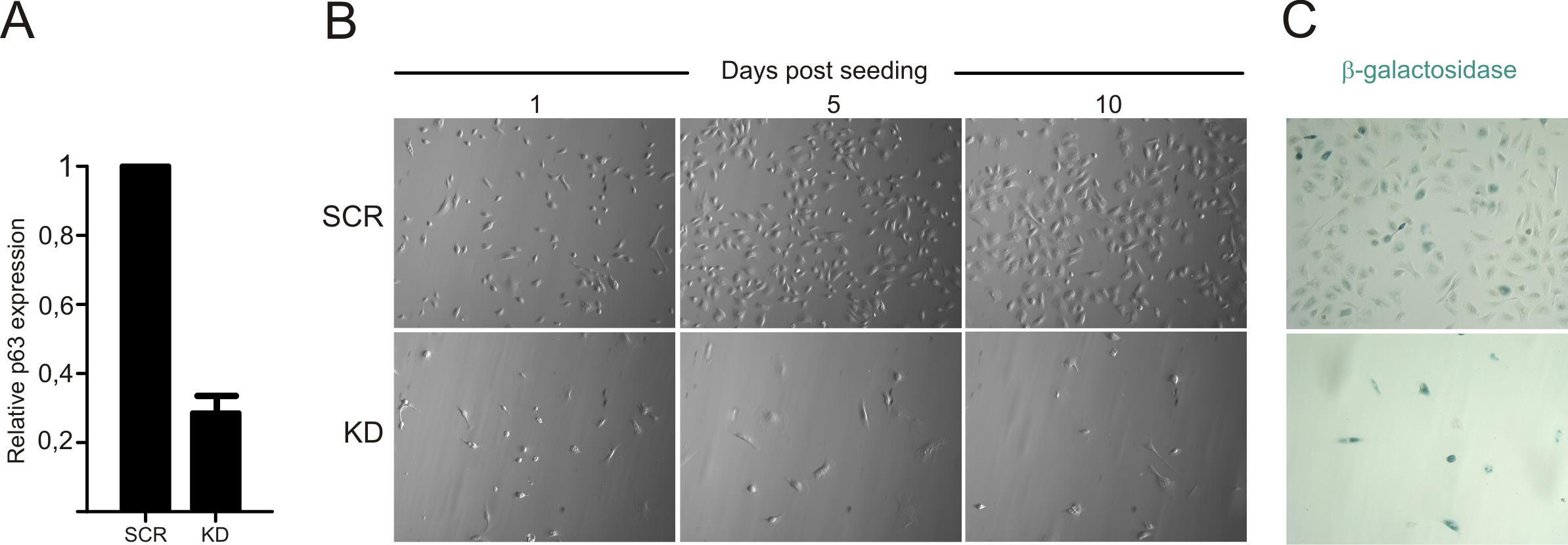

Supplement: Figure S2 — Primary bronchial cells enter growth arrest following knockdown of p63. Quantitative real time PCR shows a 71,5% knockdown of p63 in bronchial basal cells compared to scrambled vector (a). The p63 knock down cells did not proliferate when re-seeded following puromycin selection but cells infected with scrambled vector did (b). The surviving p63 knockdown cells stained positive for β-galactoside at day p10 (c). (TIF) [file pone.0088683.s002.tif]

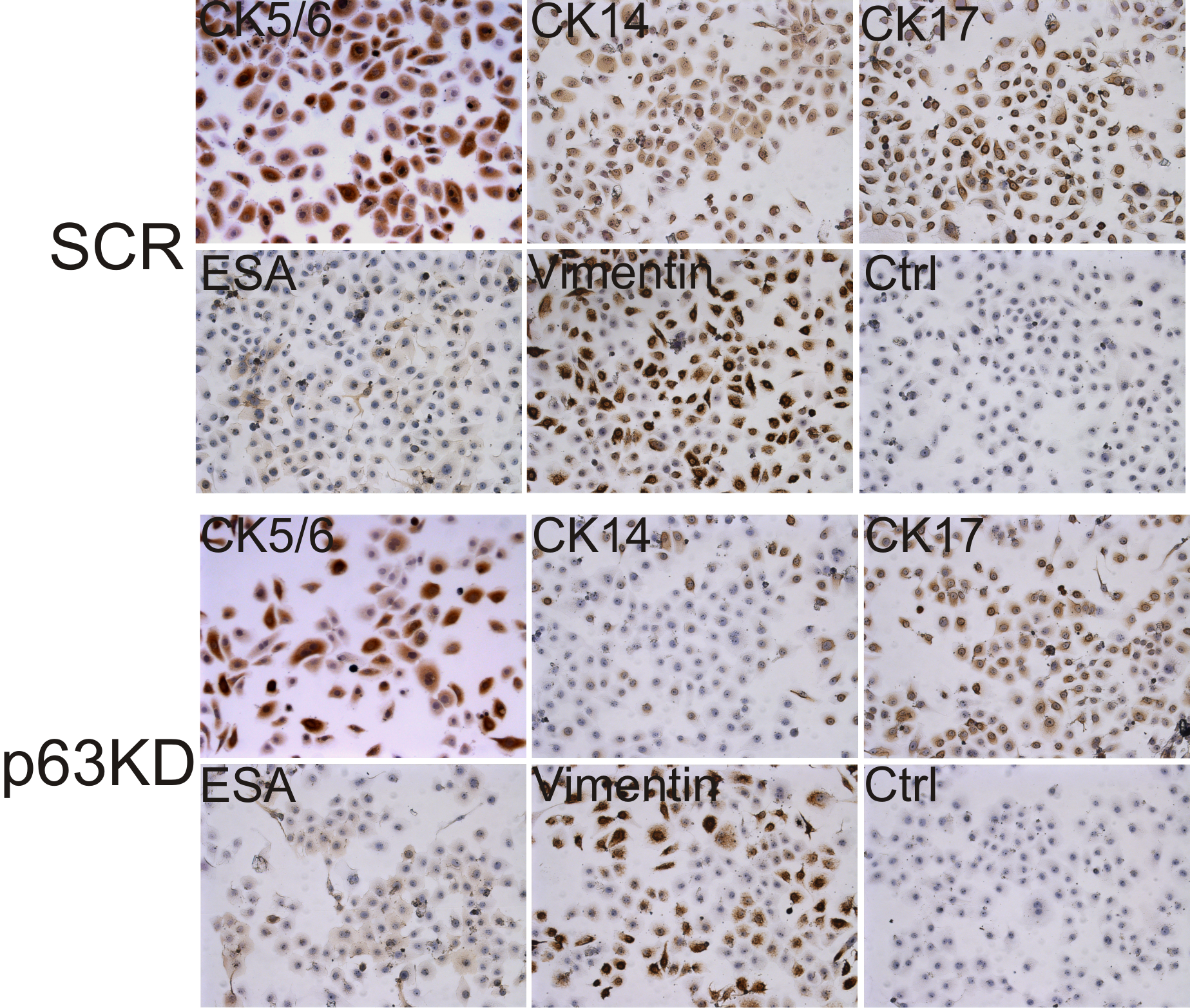

Supplement: Figure S3 — Expression of selected epithelial markers following knockdown of p63 in VA10 cells. DAB staining on VA10Scr and VA10p63kd cells shows downregulation of CK5/6, CK14 and Vimentin, upregulation of ESA and no difference in CK17 expression. The data shown represent results from two independent experiments that yielded similar results. (TIF) [file pone.0088683.s003.tif]

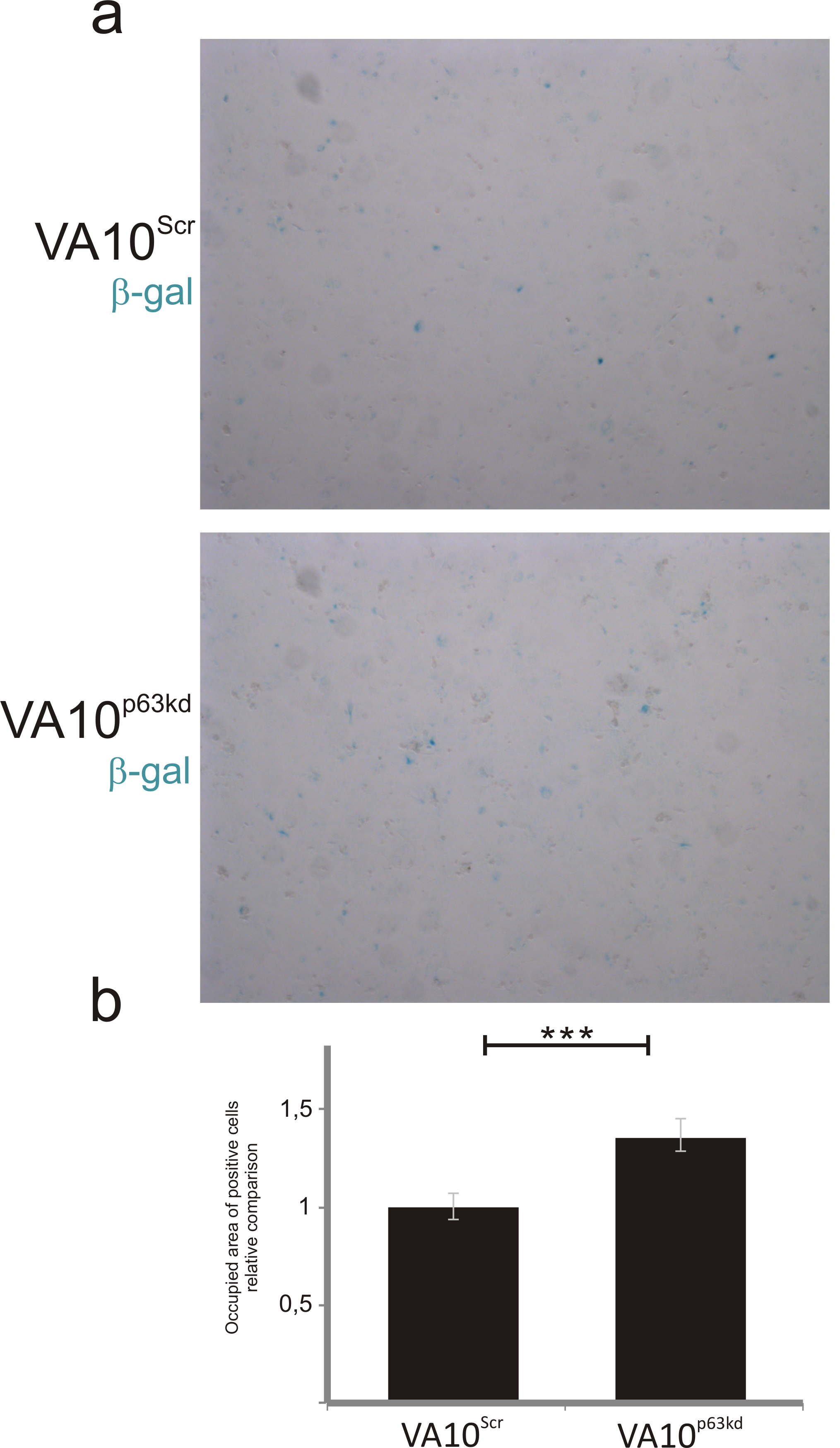

Supplement: Figure S4 — Low portion of VA10Scr and VA10p63kd cells are senescent at early confluency. β-galactosidase staining reveals low levels of senescent cells at confluency of VA10Scr (upper) and VA10p63kd (lower) cells (a). When compared, VA10p63kd cells show a 35% increase in senescence compared to VA10Scr (b).Error bars represent standard error of the means. Scale bars 50 µm. ***p≤0.001. (TIF) [file pone.0088683.s004.tif]

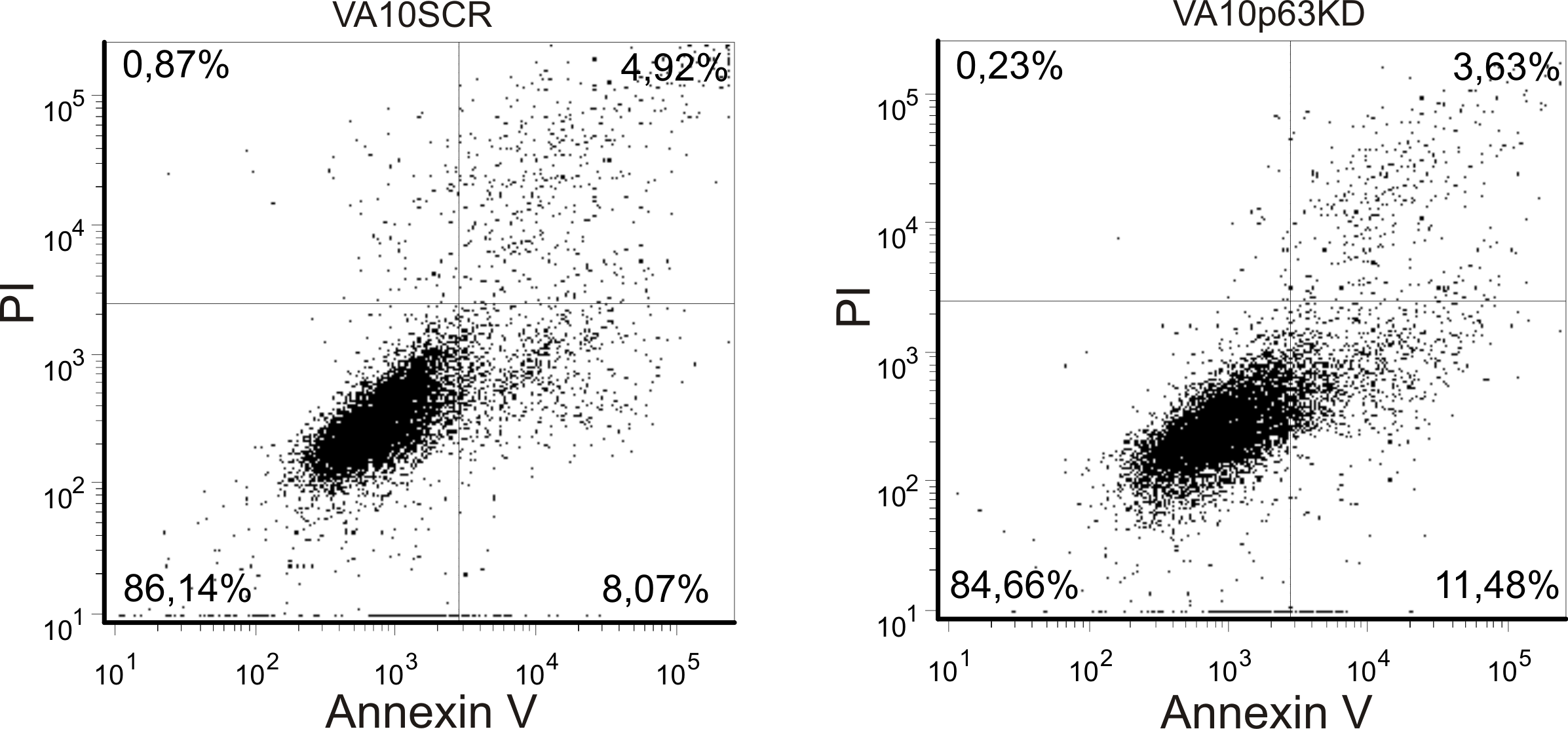

Supplement: Figure S5 — Knockdown of p63 does not affect apoptosis in monolayer VA10 cells. FACS analysis of Annexin V and PI stainings on confluent monolayer VA10Scr and VA10p63kd cells show similar portions of cells in early (Annexin high, PI low) and late (Annexin hign, PI high) apoptosis. Inset numbers represent percentage of each population in the quadrants. The data shown are represent results from two independent experiments that yielded similar results. (TIF) [file pone.0088683.s005.tif]

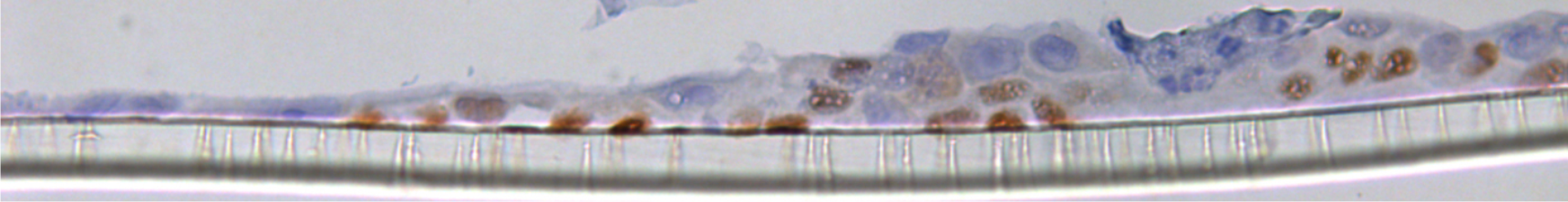

Supplement: Figure S6 — Rare patches of p63 positive cells are found in VA10p63kd epithelium. When VA10p63kd cells are cultured in ALI culture, rare patches of p63 positive cells can be found. These patches are able to form stratification and apical cells are p63-negative. (TIF) [file pone.0088683.s006.tif]
